# Maternal Folic Acid Deficiency Is Associated to Developing Nasal and Palate Malformations in Mice

**DOI:** 10.3390/nu13010251

**Published:** 2021-01-16

**Authors:** Estela Maldonado, Elena Martínez-Sanz, Teresa Partearroyo, Gregorio Varela-Moreiras, Juliana Pérez-Miguelsanz

**Affiliations:** 1 Departamento de Anatomía y Embriología, Facultad de Medicina, Universidad Complutense de Madrid, 28040 Madrid, Spain; elenamar@ucm.es (E.M.-S.); jperezm@ucm.es (J.P.-M.); 2 Grupo UCM de Investigación nº 920202 “Investigación en Desarrollo del Paladar y Fisura Palatina. Desarrollo Craneofacial”, Facultad de Odontología, Plaza de Ramón y Cajal, 3, 28040 Madrid, Spain; 3 Departamento de Ciencias Farmacéuticas y de la Salud, Facultad de Farmacia, Universidad San Pablo-CEU, CEU Universities, Urbanización Montepríncipe, Alcorcón, 28925 Madrid, Spain; t.partearroyo@ceu.es (T.P.); gvarela@ceu.es (G.V.-M.); 4 Grupo USP-CEU de Excelencia “Nutrición para la vida (Nutrition for life)”, ref: E02/0720, Alcorcón, 28925 Madrid, Spain; 5 Grupo UCM de Investigación nº 911308 “Mecanismos Moleculares Cronobiológicos”, Plaza de Ramón y Cajal, s/n, 28040 Madrid, Spain; 6 Instituto de Investigación Sanitaria del Hospital Clínico San Carlos (IdISSC), C/ del Prof. Martín Lagos, s/n, 28040 Madrid, Spain

**Keywords:** nasal region, palate, congenital abnormalities, maternal folic acid-deficient diet

## Abstract

Craniofacial development requires extremely fine-tuned developmental coordination of multiple specialized tissues. It has been evidenced that a folate deficiency (vitamin B_9_), or its synthetic form, folic acid (FA), in maternal diet could trigger multiple craniofacial malformations as oral clefts, tongue, or mandible abnormalities. In this study, a folic acid-deficient (FAD) diet was administered to eight-week-old C57/BL/6J female mouse for 2–16 weeks. The head symmetry, palate and nasal region were studied in 24 control and 260 experimental fetuses. Our results showed a significant reduction in the mean number of fetuses per litter according to maternal weeks on FAD diet (*p* < 0.01). Fetuses were affected by cleft palate (3.8%) as well as other severe congenital abnormalities, for the first time related to maternal FAD diet, as head asymmetries (4.6%), high arched palate (3.5%), nasal septum malformed (7.3%), nasopharynx duct shape (15%), and cilia and epithelium abnormalities (11.2% and 5.8%). Dysmorphologies of the nasal region were the most frequent, appearing at just four weeks following a maternal FAD diet. This is the first time that nasal region development is experimentally related to this vitamin deficiency. In conclusion, our report offers novel discoveries about the importance of maternal folate intake on midface craniofacial development of the embryos. Moreover, the longer the deficit lasts, the more serious the consequent effects appear to be.

## 1. Introduction

Craniofacial development is a complex process that begins with the participation of the frontonasal prominence and the pharyngeal arches in the earliest stages. Simultaneously, formation of the face (externally) and the nasal and oral cavities (internally) proceeds along the embryogenesis. Specifically, the nasal cavities are derived from the frontonasal prominence (including the paired lateral and medial nasal processes) and the palate develops from the maxillary prominences of the first pharyngeal arch [1]. As the nasal cavity forms, the nasal septum divides it in two and the palate separates it from the oral cavity (Figure S1). The merge of the medial nasal processes results, on the one hand, the nasal septum and, on the other, an intermaxillary segment from which the primary palate will be formed. For its part, the secondary palate is formed by the union, in the midline, of the palatal shelves developing from the medial aspect of the maxillary prominences [1]. Such structures are formed by mesoderm and neural crest cells (NCCs) that migrate from the rostral midbrain. It is also known that there are many important signaling regulators in the migration and differentiation of NCCs [2]. When any alteration in the developmental process occurs, the appearance of congenital craniofacial malformations may occur due to genetic abnormalities, environmental factors, or a combination of both.

Environmental factors such as vitamin B complex deficiencies may cause developmental disorders. It has been previously shown by our group that a folate deficiency (vitamin B_9_), or its synthetic form, folic acid (FA), in maternal diet may trigger multiple craniofacial malformations as oral clefts [3,4] or tongue and mandible abnormalities [5,6]. Moreover, it seems that FA supplementation is beneficial in reducing abnormalities such as oral clefts [7,8,9].

Orofacial clefts are the most common craniofacial malformations [3,4], therefore most of the folate deficiency-related studies and its consequences or supplementation effects have been focused on them [8,10]. However, there are many other congenital malformations that appear repeatedly in multiple syndromes, such as high-arched palate (HAP) [11], and there are no studies about the potential related role of folate status in the etiology and prevention. Even in hemifacial microsomia, which is the most common craniofacial malformation after oral clefts [3] with potential asymmetrical development of the craniofacial structures [12], there is no evidence on the role of the vitamin. This malformation is associated with an abnormal nasal region and, occasionally, palatal alterations as well. In addition, there is evidence that the nasal septum acts as a key growth center influencing the embryonic midfacial development as a whole [13,14,15]. In the present work, we therefore attempted to study the head and nasopalatine regions according to weeks on a maternal folic acid-deficient (FAD) diet.

## 2. Materials and Methods

### 2.1. Animals and Diet

The animals and diets used have been already successfully used in previous studies by our research group [16,17]. Specifically, these are similar as in previous studies [5,6]. Female mice mated only one night (12 h maximum) with a male and the following day was considered E1 (day 1 of gestation). The females began to be fed the FAD diet at E3, because the fetuses had to be removed at E17. Therefore, 14 days (2 weeks) had passed on the FAD diet when the pregnant females were sacrificed. The same method was applied for the other weeks. That is, the females were the ones who suffered this deficit in those weeks and the effects were reflected in the fetuses.

Briefly, a total of 45 eight-week-old C57/BL/6J female mice (Harlan Laboratories, Barcelona, Spain) were divided in two groups based on the experimental diet administered, only modified in its FA content as follows: control diet group (2 mg FA/kg diet, SAFE-DIETSA04/03, Panlab, Barcelona, Spain) and maternal FAD diet group (0 mg FA/kg diet + 1% succinylsulfathiazole, Harlan Laboratories, TD02490). Mice were fed their respective diets ad libitum for 2, 4, 6, 8, 10, 12, 14 or 16 weeks and were immediately euthanized (Table 1). Manipulation of the animals was performed following the European Union Normative (2003/65/CE). The experimental protocol used was reviewed and ethically approved by the Animal Welfare Ethics Committee of the Hospital Clínico San Carlos of the Universidad Complutense of Madrid (Code 08/19-18; 2009).

### 2.2. Morphological Study

At gestational day 17 (E17), pregnant female mice were killed by cervical dislocation, and fetuses were removed by caesarean section, placed in cold phosphate-buffered saline, and processed as previously described [5,6]. Briefly, the fetus heads were fixed, paraffin embedded, and 7 microns-thick sectioned and haematoxylin-eosin stained. Sections were photographed using a Nikon Eclipse Ci microscope with camera which included a NIS Elements F imaging software (Nikon Corp., Tokyo, Japan). For the highest magnification, a Nikon Eclipse Ti microscope and Nikon DS-Fil digital camera were used. The head, nasal cavity, and palate were analyzed.

### 2.3. Head and Nasopalatine Region Measurements

Comparable coronal sections of heads from control and experimental fetuses were selected as described in previous works [5,6]. Measures were performed using the image processing program Image J (National Institutes of Health (NIH), Bethesda, MD, USA, Department of Health and Human Services, Washington, DC, USA).

**Figure 1:**
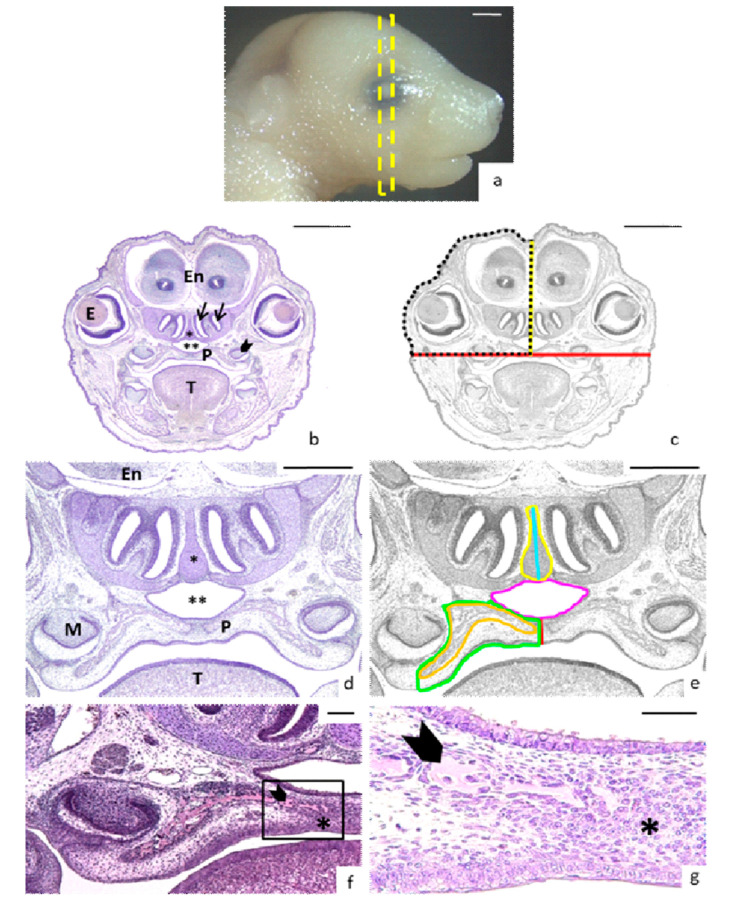
Head and nasopalatine region measurements. (**a**) Lateral view of a control head E17-fetus. The yellow rectangle crossing the eye indicates the block of sections selected for measurements. (**b**) Coronal section showing the landmarks for measurements: The nasal meatus (arrows), the eyes (E) and the developing molars (arrows heads) (En: encephalon; P: palate; T: tongue; asterisk: nasal septum; double asterisk: nasopharynx). (**c**) Description of head measurements on the same black and white image: width (red line), height from palate to top of the head (yellow line) and area of the half head (discontinuous black line). (**d**) Magnification of the nasopalatine region (T: tongue; P: palate; M: developing molar; double asterisk: nasopharynx; asterisk: nasal septum; En: encephalon). (**e**) Description of nasopalatine region measurements on the same black and white image: thick of the palate (red line); area of the half of the palate (green line); area of the bone tissue inside the palate area (orange line), area of the nasopharynx (pink line), area (yellow line) and height (blue line) of the nasal septum. (**f**) Magnification of the right half palate: bone tissue (arrowhead) and the osteogenic condensation (asterisk). (**g**) Magnification of the rectangle marked in (**f**): bone tissue (arrowhead) and the osteogenic condensation (asterisk). Scale bars: (**a**–**c**) 1 mm; (**d**–**e**) 500 µm; (**f**) 100 µm; (**g**) 50 µm.

For head measures, sections were chosen where the developing molar, eyes, and two nasal conchae were visible (Figure 1a–b). The width of the head was obtained drawing a horizontal line on the upper point of the palate vault connecting the developing molars (Figure 1b–c). The head height was obtained drawing a vertical line from the middle of the palate to the top of the head passing between olfactory bulbs of the encephalon (Figure 1c). The half head area comprised the surface between these horizontal and vertical lines and another which followed the external surface of the head (Figure 1c). To study the asymmetry of the head, the surface of the right side was compared with that of the left side. The total head area was obtained by adding the right and left areas.

For the measurement of the thick of the palate, a vertical line was drawn in the middle of the palate (Figure 1d–e). In cases of cleft palate, the thickness was registered as 0 µm. For the measurement of palate area, the lower limit was the oral epithelium until the developing molar, the external limit was the border between the developing molar and the inner bone until the corner of nasopharynx, continuing the nasopharyngeal epithelium superiorly until the internal limit (Figure 1e). The right and left measured areas were added together to obtain the palate total area.

The bone tissue surface was measured inside the palate area because the formed bone was distinguishable from the osteogenic condensation, which was a mesenchymal cell condensation before osteogenesis. The bone tissue resembled a pink-stained sea sponge, while the mesenchymal cells appeared condensed around it with the nuclei very close and was stained purple (Figure 1f–g). In some cases, the bone tissue cells appeared separated into islands or aggregates (Figure 1f–g) which were measured and added to obtain total bone tissue area on each side. The total bone area was obtained by adding its right and left areas.

The nasopharynx was an air passage over the palate, whose area was also measured (Figure 1d–e). The nasal septum area and height, which is located above the nasopharynx, was also measured (Figure 1d–e).

The measurements made reflect the original data corresponding to the histological sections without taking into account the shrinkage of tissues during processing. Three continuous sections from each embryo were measured in all cases.

### 2.4. Statistical Analysis

Values are expressed as median (interquartile range) per group. Variables were tested for normality using the Shapiro–Wilk test. Statistical differences between cases and control groups were analyzed by the Kruskal–Wallis Test and the Dunn to adjust for multiple comparison and adjust the *p* value with Bonferroni correction (altogether 36 comparisons), and Fisher’s exact test to find whether the proportions of mothers with palatal and nasal malformations fetuses are different from values of mothers without palatal and nasal malformed fetuses. Differences were considered significant at *p* < 0.05.

Spearman’s rank order correlation (Rho) was used for the total sample by variables. Correlation levels were classified according to the following categories: poor (Rho < 0.00), light (Rho = 0.00–0.20), fair (Rho = 0.21–0.40), moderate (Rho = 0.41–0.60), good (Rho = 0.61–0.80) and practically perfect (Rho = 0.81–1.00) [18]. All analysis were performed using the SPSS v.24.0 program (IBM Corp., Armonk, NY, USA).

## 3. Results

### 3.1. General Outcomes

**Figure 2:**
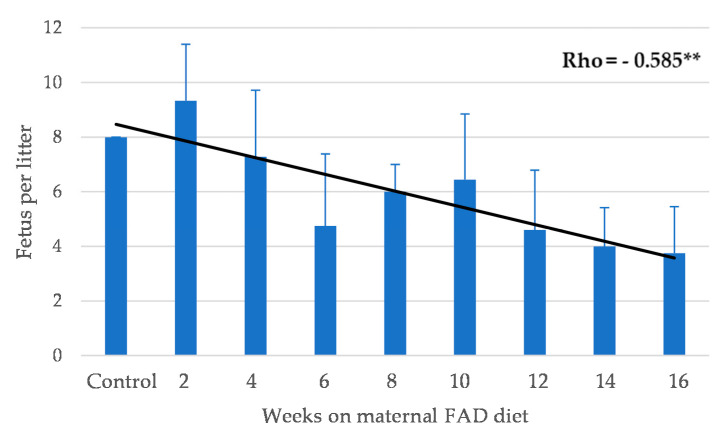
Mean number of fetuses per litter according to weeks on maternal folic acid deficient (FAD) diet. ** *p* ≤ 0.01 (Spearman’s rho test).

A total of 284 fetal heads from control (*n* = 24) and experimental fetuses (*n* = 260) were analyzed for the presence of malformations (Table 1, Table 2, and Table S1). The mean of fetuses per mother was analyzed and an inversely significant association between weeks on maternal FAD diet and the mean offspring per mother was found (Figure 2).

**Figure 3:**
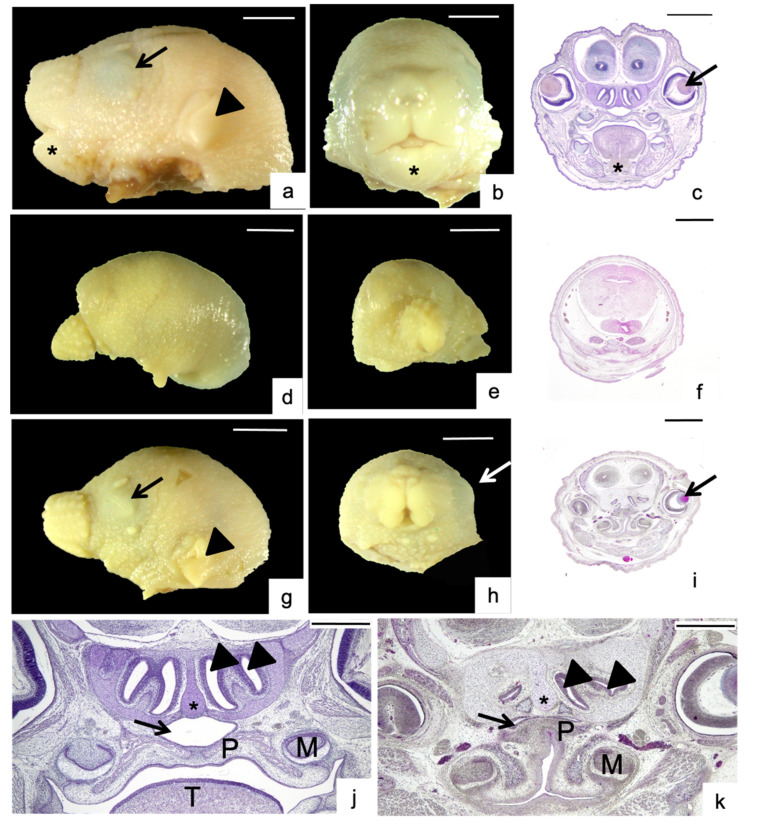
Head and palatine malformations. Control fetus head: lateral view (**a**), frontal view (**b**) and coronal section (**c**). Arrowhead: ear. Arrow: eye. Asterisk: mandible. Severely malformed fetus: lateral view (**d**), frontal view (**e**,**f**) coronal section. The eye, ear and mandible were not developed, the palate is not recognized. Malformed fetus head: lateral view (**g**), frontal view (**h**) and coronal section (**i**). This case showed asymmetrical head and high arched palate (HAP), lack of mandible and tongue. Arrowhead: ear. Arrow: eye. Magnification of nasopalatine region of control fetus (**j**): the palate (P) showed a form of truncated pyramid, the nasopharynx was like lips and they were two nasal meatus on each side of the nasal septum. Arrow: nasopharynx. Arrowheads: nasal meatus. Asterisk: nasal septum. Magnification of a malformed fetus (**k**) with HAP palate, aglossia and head asymmetry because the meatus dysmorphology. M: developing molar. T: tongue; Scale bar: (**a**–**i**) 1 mm; (**j**–**k**) 500 µm.

Measurements were not possible in seven severely malformed fetuses (2.7% of total) because the structures were not distinguishable (Table 2 and Figure 3d–f). These tremendous anomalies appeared from weeks 6 to 16, with the maximum after ten weeks on maternal FAD diet.

Therefore, we decided to study the number of mothers who had fetuses with malformations according to the week’s deficiency in FA (Table 3). We observed that when mothers remained more than 8 weeks on FAD diet the percentage of malformations in fetuses (84.0%) was significantly higher than the control mothers (0.0%) (*p* < 0.01, Fisher’s exact test).

### 3.2. Morphological Analysis

**Figure 4:**
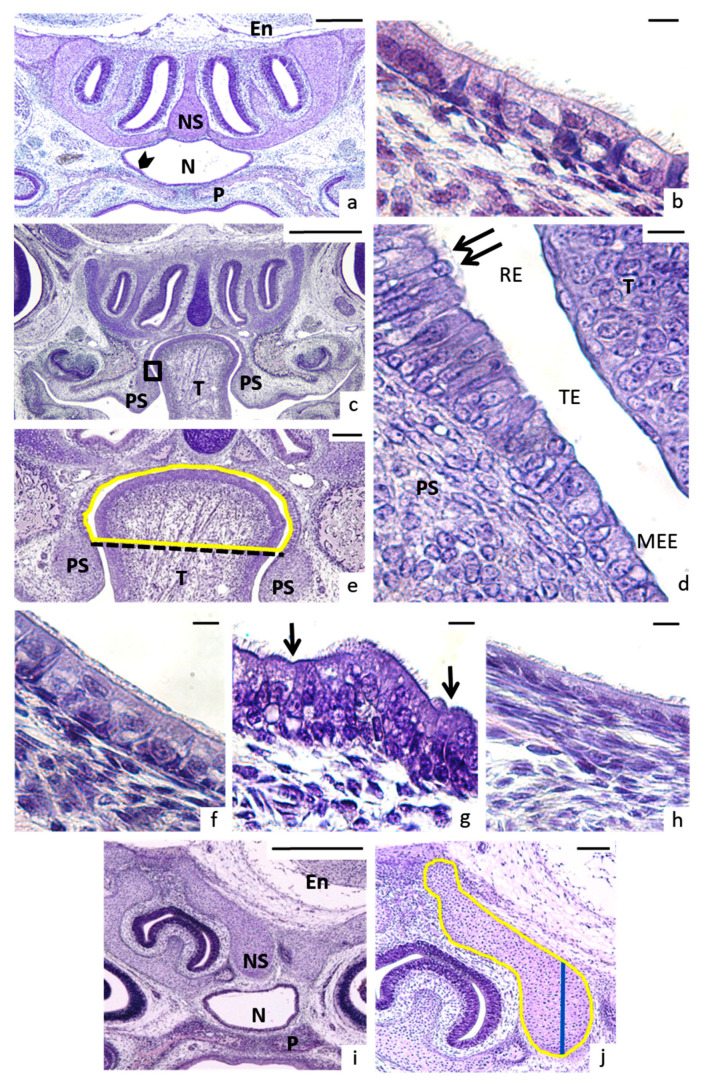
Nasopalatin region. (**a**) Coronal section of a control fetus: the nasopharyngeal duct (N) was placed between palate (P).and the nasal septum (NS), which was below the encephalon (En). Arrowhead marked the area of interest. (**b**) Magnification the control respiratory epithelium with the columnar and ciliated cells. (**c**) Malformed fetus with the tongue (T) placed between palatal shelves (PS). (**d**) Magnification of the rectangle showing the transition epithelium (TE) among respiratory (RE) to medial edge epithelium (MEE). Arrows: cilia. (**e**) Measurement of nasopharynx area (yellow line) in cleft palate. The lower limit was an imaginary line (discontinuous black line) drawn on the transitional epithelium in clef palate fetuses. (**f**) Anomalous nasopharyngeal cilia. (**g**) Arrows pointed lack of cilia in some cells. (**h**) The cells were not columnar, and cilia seemed to be atrophied. (**i**) Aberrant shape of the nasal septum (NS) and nasopharyngeal duct (N). The palate (P) was normal. En: Encephalon. (**j**) Magnification of altered nasal septum showing how the area was measured (yellow line) the height of the septum (blue line). Scale bars: (**a**,**c**,**i**) 500 µm; (**b**,**d**,**f**–**h**) 10 µm; (**e**,**j**) 100 µm.

Malformations affected the nasopalatine regions, as shown in Figure 3 and Figure 4 and Table 1, Table 2, Table 3 and Tables S1–S3. In control fetuses, the right and left side of the head were comparable. Asymmetry was designated when a lack of symmetry, especially in parts and organs that appear duplicated in the head and, which are usually similar, were observed (Figure 3g–k): twelve cases (4.6% of total) between 8 to 12 weeks on maternal FAD diet (Table 2 and Table S3).

Malformations of the palatal region affected nineteen cases, starting at week 6 on maternal FAD diet up to the end of the study, with the highest incidence appeared in week 10. The palate had a truncated pyramid form and two types of dysmorphologies were found. In high arched palate (HAP) the palate was fused but unusually high and narrow, like an inverted “V”, with the molars closer than control. HAP affected 9 fetuses (3.5% of total) from 6 to 16 weeks. This alteration was always associated to aglossia (absence of tongue) (Figure 3j,k, Table 2). Cleft palate occurred when the palatal shelves had not merged (Figure 4a,c): it was shown in ten fetuses (7.3% of total) from 8 to 16 weeks.

The nasal region entails diverse and complex structures. The nasopharyngeal duct is the wide air channel placed between palate below and nasal septum above, which looked like lips (Figure 4a). Nasopharyngeal epithelium had columnar ciliated cells. Lateral to the septum, two air passages on each side constituted the nasal meatus (Figure 3j,k and Figure 4a,b). These structures could be affected in diverse ways (Table 2, Figure 4), being the nasopharynx duct shape affected in the highest proportion (15.0% of total), followed by cilia (11.2%), nasal septum (7.3%), and nasopharynx epithelium (5.8%). See Figure 4 for details. These dysmorphologies were also the first to occur, at 4 weeks on maternal FAD diet and could appear either isolated or accompanying other malformations (Table 1, Table 2 and Table S2; Figure 3 and Figure 4).

### 3.3. Head, Palate, and Nasal Measurements

Although we attempted to analyze 284 fetuses (Table 1 and Table S1), finally a total of 217 from control (*n* = 15) and experimental fetuses (*n* = 202) were measured (between 53.6% and 94.1% depending on the weeks under deficiency) since some samples did not meet sufficient histological quality criteria to be included (Tables S2 and S3).

The maternal FAD diet only affected right half, left half, and total palate area at 16 weeks (Table S2) when compared to the control group. Surprisingly, the nasopharyngeal area and the area and height of the septum nasal measurements were not significantly different, even though they were reduced by more than 38.8% in nasopharynx duct area respect to control group (see Table S2 for details).

Head measurements (width; height; right half, left half, and total head area) were all significantly reduced up to 37%, starting after eight weeks on maternal FAD diet until the end of the study (see Table S3 for details).

**Figure 5:**
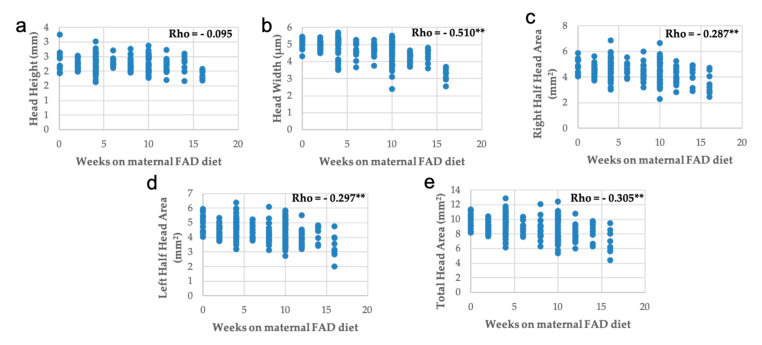
Correlations between head measurements with weeks on maternal FAD diet. (**a**) Head width; (**b**) head height; (**c**) right half head area; (**d**) left half head area and (**e**) total head area. ** *p* ≤ 0.01; Spearman’s Rho.

Correlations between head measurements and weeks on maternal FAD diet were inversely and significantly correlated, except for the height of the head (Figure 5).

**Figure 6:**
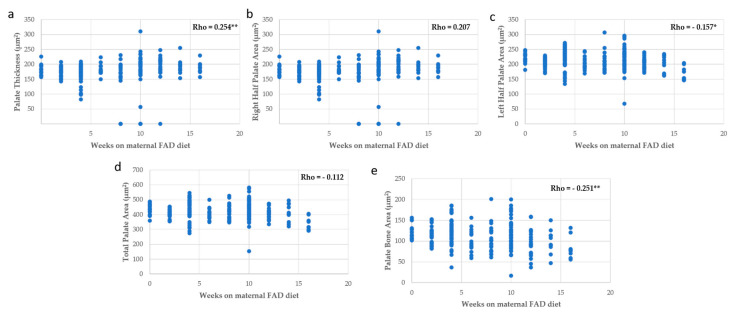
Correlations between palate measurements with weeks on maternal FAD diet. (**a**) Palate thickness; (**b**) right half palate area; (**c**) left half palate area; (**d**) total palate area and (**e**) palate bone area. * *p* ≤ 0.05; ** *p* ≤ 0.01.

**Figure 7:**
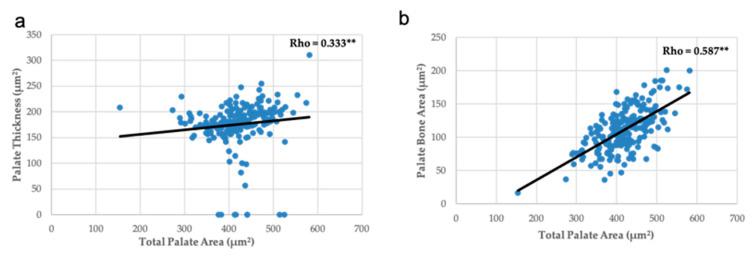
Correlations between palate measurements and total palate area. (**a**) Palate thickness and (**b**) palate bone area. ** *p ≤* 0.01; Spearman’s Rho.

**Figure 8:**
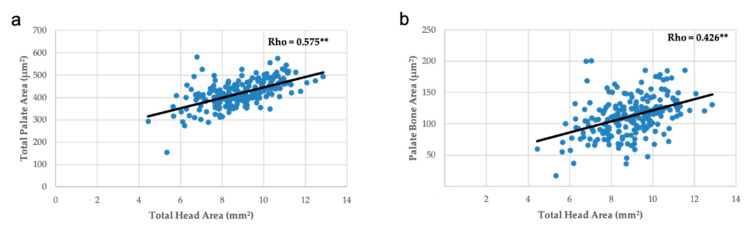
Correlations between palate measurements and total head area. (**a**) Total palate area and (**b**) palate bone area. ** *p ≤* 0.01; Spearman’s Rho.

Measurement of palate area was inversely and significantly correlated with the number of weeks on maternal FAD diet, but the thickness of palate was directly correlated (Figure 6). The thickness and the bone area showed a proportional variation with the palate area (Figure 7), like the area of the bone and the thickness of palate with the head area (Figure 8).

**Figure 9:**
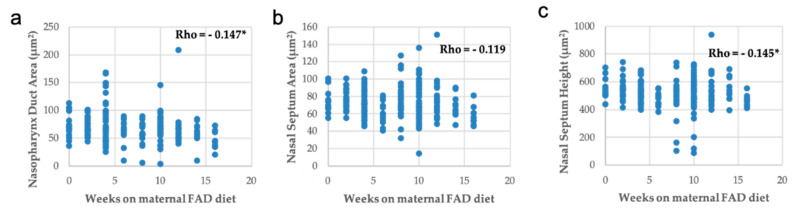
Correlations between nasal measurements with weeks on maternal FAD diet. (**a**) Nasopharynx duct area, (**b**) nasal septum area and (**c**) nasal septum height. * *p ≤* 0.05; Spearman’s Rho.

**Figure 10:**
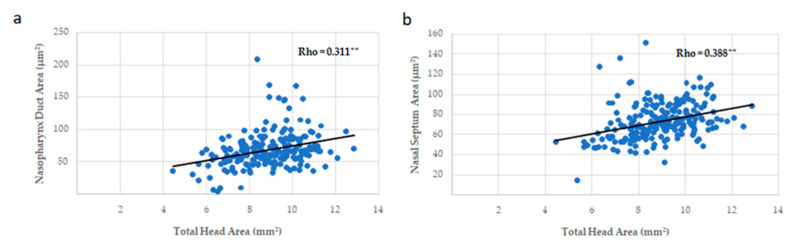
Correlations between nasal measurements and total head area. (**a**) Nasopharynx duct area and (**b**) nasal septum area. ** *p ≤* 0.01; Spearman’s Rho.

Lastly, the correlations between nasal measurements and the number of weeks under maternal FAD diet presented statistical significance, except for the nasal septum area (Figure 9). Moreover, a positive significant correlation with head area was observed (Figure 10).

In summary, the increase in the number of weeks on a FAD diet showed a significant positive correlation only with the thickness of the palate (Figure 6a); a very significant negative correlation with the mean fetuses per litter (Figure 2), the width of the head and the right, left and total head areas (Figure 5). A significant negative correlation was observed between the left palate area (Figure 6c), bone tissue area (Figure 6e) and the nasopharyngeal duct area (Figure 9a), as well as the height of the nasal septum (Figure 9c). All correlations between the total palate area (Figure 7) and the total head area (Figure 8 and Figure 9) with bone, nasopharyngeal duct and nasal septum were positive and very significant, decreasing their values as the weeks on maternal FAD diet progressed.

## 4. Discussion

In the present study, we analyzed the different anomalies that arose in the nasal and palate regions of mouse fetuses due to a FA lacking diet consumed by pregnant mothers during different weeks. Maternal hepatic folate concentrations showed the effectiveness of our experimental model, as we have previously reported [5,6]. Specifically, folate levels were reduced after two to four weeks on the FAD diet compared to control group and over the period from six to sixteen weeks were significantly lower (*p ≤* 0.01) than for the two to four weeks FAD diet animals and the control group.

For the first time we show evidence that a maternal FAD diet caused a marked impairment of nasopalatine region, whereas an adequate maternal FAD diet had a powerful protective role in preventing nasal and palatine dysmorphologies.

The first effect observed was the decline in the number of fetuses as the weeks of maternal FAD diet increased but also the number of affected fetuses in proportion to the size of the litters. These data were consistent with epidemiological studies where low folate levels and high plasma homocysteine levels (hyperhomocysteinemia) were associated with increased miscarriage risk [19,20], which could be due to low folate directly or to the hyperhomocysteinemia associated with vascular alterations that affect the placenta [21]. Furthermore, folate receptor ***Mthfr*** knock out mutant embryos showed multiple malformations, development delay and numerous resorptions [22], decreasing litter size in the same way as in our experimental model.

Since our interest in this study was focused on the nasopalatine region, we decided to perform a series of measurements on the head related to this area. The measurements of the nasopalatine region showed a reduction in size as the maternal FAD diet weeks increased, which was very significant from week 12. This reduction in size has been also observed in previous studies by our group [5,6] and other authors [23,24,25].

In addition, the performed series of measurements on nasal and palatal structures displayed a proportional, harmonic, and significant reduction related to what occurred in the head. The only structure that seemed to increase in size with increasing weeks on FAD diet was the thickness of the palate, which may be explained because the width and the area of the head decreased significantly but not the palate area. Therefore, the same palate area had to be kept in a diminishing and smaller space, which could cause a remodeling and accommodation in a vertical arrangement, especially when no significant variation in head height was observed.

Regarding the malformations found, we already expected that a percentage of the fetuses whose mothers underwent the FAD diet would suffer from cleft palate and, indeed, we observed 3.5% of cases with this anomaly. These results were anticipated by previous studies on the relationship of folate and orofacial clefts, which described that low folate levels in the maternal diet caused an increase in the incidence of clefts [26] and the beneficial effect of FA administration [27] on this dysmorphologies, although there was controversy in what the exact relationship between both factors was [8]. Surprisingly, we also found a similar percentage (3.8%) in another scarcely known and therefore less studied palate anomaly, the HAP. This malformation is associated to others defects in various human syndromes (Crouzon, Turner, Vand der Woude) [11], as well as in the *Fuz* mutant mice [28], but it is associated for the first time with a maternal FAD diet in the present study.

In addition, as a novelty, we found anomalies related to the nasal region such as the alteration of the shape of the nasopharyngeal duct. This alteration was evident in the cases with CP or HAP or associated to cases of asymmetry as part of the spectrum of hemifacial microsomy, which is another of the most common craniofacial malformations along with orofacial clefts [3], but it had not been previously described in isolation, as we were able to notice in several malformed fetuses. In the cases of asymmetry, half of the head developed differently from the other half, leading to abnormalities such as the aforementioned unusual shapes of the nasopharyngeal duct or the nasal septum. As we did not observe a significant difference between the size of the right and the left area, we conclude that the variance was due to the speed with which the structures were formed. Hemifacial microsomy has been described as arising from abnormalities in the development of the 1st and 2nd pharyngeal arches [29]. These arch anomalies could also alter the development of the jaw, affected by maternal FAD diet as we analyzed in previous works [5,6], as well as the development of the palate observed in the present study.

We found anomalies in the shape of the nasal septum, although the differences in area and height compared to the control group were not statistically significant. The shape of the nasal septum was always altered in asymmetries and CP. Analysis of the nasal septum is important since its correct development seemed to be essential for the nasofacial skeleton to develop properly [30]. In fact, there was evidence in C57 mice that the growth of the nasal septum was correlated with the increase in the dimensions of the palate and nasal bones [14]. Furthermore, the key role of nasal septum during development could have a higher influence in mammals with shorter faces such as humans [31]. It is important to notice that both, in the case of asymmetries and in the alteration of the nasopharyngeal duct and the nasal septum described in the present work, it is the first time that FAD has been related to these dysmorphologies.

Another novel finding recorded in this study was the atrophy of the cilia and the disruption of the nasopharyngeal epithelium due to the maternal FAD diet. We did not find evidence of these anomalies in other studies, so it may be a future area of study. The only evidence of such was a study where its authors observed that when folate receptors were overexpressed (in deficit situations) there was a decrease in cellular proliferation of some structures as the olfactory epithelium [32]. These dramatic alterations of the epithelium and the shape of the nasopharyngeal duct were the first to emerge, after only four weeks of FAD. These findings reinforce what has been described previously [16], in which no fissures appeared at two weeks on maternal FAD diet, but alterations in the basic mechanisms of palate development were already observed. Therefore, it is worth noting that microscopic alterations could exist early from just two weeks on maternal FA deficiency, although most of the most serious defects begin to appear after six weeks of deficit, adding tremendous value to the importance of acquiring the correct levels of FA during the development stages. This fact could be somewhat related to the accumulation of folates in the maternal liver [16,17]. These studies explained how there was a slight decrease in hepatic folate during the first four weeks without FA in the diet, but the decrease was very significant from six weeks onwards.

The fact that not all fetuses carried malformations, and these were of multiple types and combinations, could be due to the individual genotype and would generate a variety of effects of the maternal FAD diet [33,34]. In that regard, we wish to emphasize that great attention was paid to avoiding inbreeding, which could be a source of malformations and could distort the results.

The sum of the malformations with the cases described as severely malformed highlights again the importance of adequate FA levels at very early stages of development when the neural crest cells begin their migration to the branchial arches and frontonasal prominence to form the head [1]. It is known that folate reduces the synthesis of nitrogenous bases [35,36], which could affect cell proliferation, very active during embryonic development. In fact, it has been observed that the blockade of folate receptors affected the cellular proliferation of the tongue [32] and that the FAD diet reduced the proliferation of the palate mesenchymal cells in development [16] and that of neural crest cells [37] in animal models.

In addition to affecting cell proliferation, it is possible that a decrease in folate levels generated an alteration in the basic signaling pathways for the correct embryonic development. In the dynamics of neural crest cells, the role of transforming growth factor beta (*TGF-β*), particularly *TGF-β2* and *β3*, had an essential role [38]. In fact, it has been proved that FAD could alter the expression of *TGF-β1* in the rat calvaria [39] as well as of *TGF-β3* in the developing of mouse palate [16]. Consequently, FAD seemed to affect the expression of these growth factors with potential functional consequences.

*TGF-β* are part of an essential signaling pathway in embryonic development as well as those involving *Shh, Wnt* and *FGF*, all considered the “master regulators” of development [40]. The importance of *Shh* and *Wnt* pathways has been known through the study of the ciliopathies, a group of diseases caused by alterations in primary cilium [41]. These organelles are essential in basic cellular mechanisms that generate the mitotic spindle directing the orientation of cell division, an important mechanism because it sets the pattern of development of tissues and organs [42]. Furthermore, the anomalies described in ciliopathies were mainly craniofacial and neural tube defects [28,43] in which nutrients modulation (e.g., folic acid) may exert an important role.

The most feasible for the genes involved in these signaling pathways affected by the FA status appears to reside in the relationship of folate metabolism with the production of essential methyl groups in the methylation process [44]. In fact, methylation of certain regions affects gene expression, so if the affected genes are as important as those involved in the *Shh* signaling pathway, *Wnt*, *FGF* or *TGF-β* anomalies described could emerge.

## 5. Conclusions

Adequate folates intakes and levels are essential, especially relevant in embryonic development, since a deficit in the maternal FA diet during the earliest stages may cause multiple craniofacial alterations in the nasal region as shown for the first time in the present study . Furthermore, the longer the deficit lasts, the more serious the consequent effects seem to be.

## Figures and Tables

**Table 1:** General outcome. Number of fetuses and incidence of malformation per week on a maternal folic acid-deficient (FAD) diet.

Weeks on Maternal FAD Diet	Mothers (*n*)	Number of Fetuses (*n*)	Nasal Malformations Only *n* (% Total)	Nasopalatine Malformations *n* (% Total)	Total Malformed *n* (% Group)
Control	3	24	0 (0.0)	0 (0.0)	0 (0.0)
2	6	56	0 (0.0)	0 (0.0)	0 (0.0)
4	7	51	13 (5.0)	0 (0.0)	13 (5.0)
6	4	19	0 (0.0)	2 (0.8)	2 (0.8)
8	5	30	5 (1.9)	5 (1.9)	10 (3.8)
10	9	58	4 (1.5)	13 (5.0)	17 (6.5)
12	5	23	7 (2.7)	1 (0.4)	8 (3.1)
14	3	12	1 (0.4)	2 (0.8)	3 (1.1)
16	3	11	1 (0.4)	2 (0.8)	3 (1.1)
**Total**	**45**	**284**	**31 (11.9)**	**25 (9.6)**	**56 (21.5)**

**Table 2:** Type and incidence of malformations per weeks on a maternal folic acid-deficient (FAD) diet.

	Asymmetry	Palatal Region Malformations		Nasal Region Malformations			
Weeks on Maternal FAD Diet	*n* (% Total; % Malformed)	HAP	CP	Nasal Septum	Nasopharynx Duct Shape	Cilia	Nasopharynx Epithelium
		*n* (% Total; % Malformed)	*n* (% Total; % Malformed)	*n* (% Total; % Malformed)	*n* (% Total; % Malformed)	*n* (% Total; % Malformed)	*n* (% Total; % Malformed)
Control	0 (0.0; 0.0)	0 (0.0; 0.0)	0 (0.0; 0.0)	0 (0.0; 0.0)	0 (0.0; 0.0)	0 (0.0; 0.0)	0 (0.0; 0.0)
2	0 (0.0; 0.0)	0 (0.0; 0.0)	0 (0.0; 0.0)	0 (0.0; 0.0)	0 (0.0; 0.0)	0 (0.0; 0.0)	0 (0.0; 0.0)
4	0 (0.0; 0.0)	0 (0.0; 0.0)	0 (0.0; 0.0)	0 (0.0; 0.0)	9 (3.5; 16.1)	12 (4.6; 21.4)	5 (1.9; 8.9)
6	0 (0.0; 0.0)	1 (0.4; 1.8)	0 (0.0; 0.0)	0 (0.0; 0.0)	1 (0.4; 1.8)	0 (0.0; 0.0)	0 (0.0; 0.0)
8	5 (1.9; 8.9)	1 (0.4; 1.8)	3 (1.2; 5.4)	6 (2.31; 10.71)	7 (2.7; 12.5)	4 (1.5; 7.1)	1 (0.4; 1.8)
10	5 (1.9; 8.9)	5 (1.9; 8.9)	5 (1.9; 8.9)	10 (3.08; 14.29)	14 (5.4; 25.0)	5 (1.9; 8.9)	4 (1.5; 7.1)
12	2 (0.7; 3.6)	0 (0.0; 0.0)	1 (0.4; 1.8)	2 (0.8; 3.6)	5 (1.9; 8.9)	5 (1.9; 8.9)	3 (1.2; 5.4)
14	0 (0.0; 0.0)	1 (0.4; 1.8)	0 (0.0; 0.0)	0 (0.0; 0.0)	1 (0.4; 1.8)	1 (0.4; 1.8)	1 (0.4; 1.8)
16	0 (0.0; 0.0)	1 (0.4; 1.8)	1 (0.4; 1.8)	1 (0.4; 1.8)	2 (0.8; 3.6)	2 (0.8; 3.6)	1 (0.4; 1.8)
**Total**	**12 (4.6; 21.4)**	**9 (3.5; 16.1)**	**10 (3.8; 17.9)**	**19 (7.3; 33.9)**	**39 (15.0; 69.6)**	**29 (11.2; 51.8)**	**15 (5.8; 26.8)**

HAP: high arched palate; CP: cleft palate. A fetus could suffer more than one abnormality.

**Table 3:** Number of mothers with malformed fetuses per week on a maternal FAD diet.

	Weeks on Maternal FAD Diet				
		0	2–6	8–16	Total
Mothers without malformed fetuses	*n* (% group)	3 ^a^ (100.0)	11 ^a^ (64.7)	4 ^b^ (16.0)	18 (40.0)
Mothers with malformed fetuses	*n* (% group)	0 (0.0)	6 ^a^ (35.3)	21 ^b^ (84.0)	27 (60.0)
Total	*n* (% group)	3 (100.0)	17 (100.0)	25 (100.0)	45 (100)

Each letter of the subscript denotes a subset of weeks categories whose column ratios do not differ significantly from each other at the 0.05 (Fisher’s exact test).

## Data Availability

The data presented in this study are available on request from the corresponding author.

## Supplementary Materials

The following are available online at https://www.mdpi.com/2072-6643/13/1/251/s1, Figure S1. Diagrams show different views illustrating progressive stages in the craniofacial development in the mouse embryo including nasal and oral cavities. Table S1. General outcome. Number and percentage of fetuses and type and incidence of malformations with respect to weeks on a maternal FAD diet; Table S2. Effects of maternal FAD diet on palate and nasal measurements; Table S3. Effects of maternal FAD diet on head measurements.
