# Supplementary material for: Maternal Folic Acid Deficiency Is Associated to Developing Nasal and Palate Malformations in Mice
Source: Nutrients. 2021 Jan 16;13(1):251. doi: 10.3390/nu13010251 (PMC7830789; doi:10.3390/nu13010251)
Supplement: Supplementary file 1 [file nutrients-13-00251-s001.zip › nutrients-1046804-Supplementary/FIGURE S1. Maldonado et al. Nutrients 2021, 13, 251.pdf]

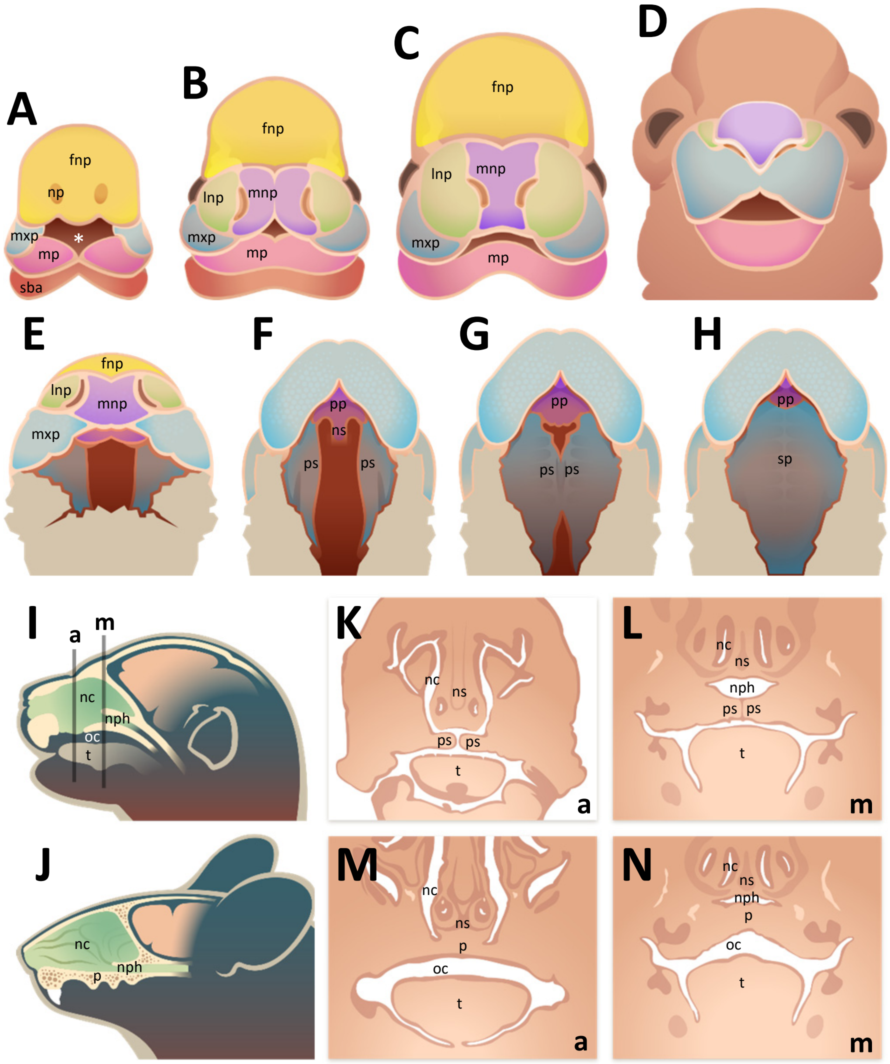

Figure S1. Representative diagrams show different views illustrating progressive stages in the craniofacial development in the mouse embryo including nasal and oral cavities. (**A**–**D**) Frontal views of the head show the advances in the development of the face. **A**, At E9.5 show the prominences surrounding the stomodeum (*) that prompt future structures, namely the frontonasal process (yellow) —from which the nasal placode arise—, the first branchial arch —divided in the maxillary processes (blue) and the mandibular process (pink)—, and the secondary branchial arch (red). **B**, At E11.5 show the nasal pits invagination and the emergence of the medial (purple) and lateral (green) nasal processes in the frontonasal process. **C**, At E12.5 show the paired medial nasal processes merged in the midline (from which the primary palate will be formed). **D**, At E14.5 show the maxillary processes fused in the midline and forming one continuous structure. It also show the maxillary processes fused with the lateral nasal processes. (**E**–**H**) Inferior views showing the stages of palatogenesis from the appearance of the palatal primordia at E11 (**E**). **F**, At E13 show the palatal shelves (blue) growing vertically and starting to elevate to become horizontal at around E14. Then, the palatal shelves midline (from which the secondary palate will be formed) grow toward each other and establish contact at the midline at around E14.5 (**G**). As shown, the palatal shelves contact first at the junction between the anterior and middle region and then proceed both anteriorly and posteriorly along the entire anteroposterior length. Finally, at E15 (**H**) palatal fusion is evident and by E15.5 the palatal shelf closure is completed. **I**,**J**, Cartoons depicting a median sagittal aspect in which the nasal septum is not been represented. **I**, Mouse embryo indicating the level of coronal sections of **K**,**L**,**M**,**N** (a, anterior —**K**,**M**—; m, middle —**L**,**N**—). **J**, Mouse adult show the definitive conformation of the nasal and oral cavities. (**K**,**L**,**M**,**N**) Schematic representation of the appearance of coronal sections. **K**, At E14.5 show the palatal shelves in a horizontal position approaching each other in the anterior region. However, in the middle region, at E14.5 palatal shelves already establish contact in the midline (**L**). At E15 the palatal shelves appear fused both in the anterior (**M**) and in the middle region (**N**). At the same time, in the anterior region the nasal septum meet the upper surface of the palate (**M**). In addition, in the middle region the nasopharynx duct is formed as an air passage over the palate (**L**,**N**). (**Abbreviations**: a, anterior region; fnp, frontonasal process; lnp, lateral nasal processes; m, middle region; mnp, medial nasal processes; mp, mandibular process; mxp, maxillary processes; nc, nasal cavity; np, nasal placode; nph, nasopharynx; ns, nasal septum; oc, oral cavity; p, palate; pp, primary palate; ps, palatal shelves; sba, secondary branchial arch; sp, secondary palate; t, tongue; white asterisk, stomodeum).

Citation: Maldonado, E.; Martínez-Sanz, E.; Partearroyo, T.; Varela-Moreiras, G.; Pérez-Miguelsanz, J. Maternal Folic Acid Deficiency Is Associated to Developing Nasal and Palate Malformations in Mice. *Nutrients* **2021**, *13*, 251. https://doi.org/10.3390/nu13010251
